# Environmental Chemicals: Evaluating Low-Dose Effects

**DOI:** 10.1289/ehp.1205179

**Published:** 2012-03-14

**Authors:** Linda S. Birnbaum

**Affiliations:** Director, NIEHS and NTP, National Institutes of Health, Department of Health and Human Services, Research Triangle Park, North Carolina, E-mail: birnbaumls@niehs.nih.gov

Around the world, large-scale biomonitoring programs have provided extensive information about human exposure to a large number of environmental chemicals (Barr et al. 2010; Bilau et al. 2008; Churchill et al. 2001; Woodruff et al. 2011). As these programs extend to look at vulnerable populations, including pregnant women, fetuses, and the elderly, our knowledge of the widespread distribution of many of these chemicals—including hundreds that have been classified as endocrine disruptors—continues to climb. However, the mere presence of a chemical in humans is not necessarily cause for concern. What is concerning is the increasing number of epidemiological studies showing associations between the concentration of these chemicals in the general population and adverse health end points (Braun and Hauser 2011; Crain et al. 2008). Although high exposures following accidental or occupational exposures to endocrine disruptors, industrial chemicals, pesticides, and pharmaceuticals have shown striking effects, epidemiological studies suggest that low doses may also be unsafe, even for populations that are not typically considered “vulnerable.”

Making connections between the exposome and risk assessment is a difficult but important venture (Paustenbach and Galbraith 2006; Rappaport and Smith 2010). Risk assessments typically examine the effects of high doses of administered chemicals to determine the lowest observed adverse effect levels (LOAELs) and no observed adverse effect levels (NOAELs); reference doses, which are assumed safe for human exposure, are then calculated from these doses using a number of safety factors. Thus, human exposures to thousands of environmental chemicals fall in the range of nonnegligible doses that are thought to be safe from a risk assessment perspective. Yet the ever-increasing data from human biomonitoring and epidemiological studies suggests otherwise: Low internal doses of endocrine disruptors found in typical human populations have been linked to obesity (Carwile and Michels 2011), infertility (Meeker and Stapleton 2010), neurobehavioral disorders (Swan et al. 2010), and immune dysfunction (Miyashita et al. 2011), among others.

For several decades, environmental health scientists have been dedicated to addressing the “low-dose hypothesis,” which postulates that low doses of chemicals can have effects that would not necessarily be predicted from their effects at high doses. More than 10 years ago, a National Toxicology Program expert panel concluded that there was evidence for low-dose effects for a select number of well-studied endocrine disruptors (Melnick et al. 2002). Now, a diverse group of scientists has reexamined this large body of literature, finding examples of low-dose effects for dozens of chemicals across a range of chemical classes, including industrial chemicals, plastic components and plasticizers, pesticides, phytoestrogens, preservatives, surfactants and detergents, flame retardants, and sunblock, among others (Vandenberg et al. 2012). Vandenberg et al. selected several examples of controversial low-dose test cases and applied an analytical weight-of-evidence approach to determine whether there was sufficient evidence to conclude that particular environmental chemicals had effects on specific biological end points. Their analysis addresses how experimental design, choice of animal strain/species, study size, and inclusion of appropriate controls affect the outcome and interpretation of studies on bisphenol A (BPA), atrazine, dioxin, and perchlorate. Their study provides important insight into the effects of environmental chemicals on health-related end points and addresses the mechanistic questions of how chemicals with hormonal activity can have effects at external doses that are often considered safe by the regulatory community.

Vandenberg et al. (2012) have also collected several hundred examples of nonmonotonic dose–response curves (representing many classes of environmental chemicals) that have been observed in cultured cells, animals, and even human populations (Vandenberg et al. 2012). Most importantly, they reviewed the voluminous endocrine literature on how and why nonlinear responses manifest at different levels of biological complexity, including the combination of competing monotonic responses (such as enhanced cell proliferation and cytotoxicity), the expression of cell- and tissue-specific cofactors and receptors, and receptor down-regulation, desensitization, and competition. Thus, the question is no longer whether nonmonotonic dose responses are “real” and occur frequently enough to be a concern; clearly these are common phenomena with well-understood mechanisms. Instead, the question is which dose–response shapes should be expected for specific environmental chemicals and under what specific circumstances.

Moving forward, studies of suspected endocrine disruptors need to include doses that result in relevant internal human levels and examine a wide range of biological end points. Dose–response studies should include a range of doses to distinguish between linear monotonic and nonmonotonic responses. Nonlinear relationships should not be dismissed. Collaborations between research scientists in academia, government, and industry should be encouraged to allow for development of more sophisticated study designs to facilitate regulatory decisions. It is time to start the conversation between environmental health scientists, toxicologists, and risk assessors to determine how our understanding of low-dose effects and nonmonotonic dose responses influence the way risk assessments are performed for chemicals with endocrine-disrupting activities. Together, we can take appropriate actions to protect human and wildlife populations from these harmful chemicals and facilitate better regulatory decision making.
